# Morphea after SARS-CoV2 vaccine^⋆^

**DOI:** 10.1016/j.abd.2022.04.010

**Published:** 2023-02-06

**Authors:** Giovanni Paolino, Matteo Riccardo Di Nicola, Nathalie Rizzo, Santo Raffaele Mercuri

**Affiliations:** a Unit of Dermatology and Cosmetology, IRCCS San Raffaele Hospital, Milano, Italy; b Surgical Pathology, IRCCS San Raffaele Hospital, Milano, Italy

Dear Editor,

Along with the increasing administration of COVID-19 vaccines, in the last months, the detection of potential adverse skin reactions has increased, highlighting the possibility that not only SARS-CoV-2 infection but also COVID-19 vaccines can induce cutaneous manifestations.^1^, ^2^ In this regard, type I hypersensitivity reactions (e.g., urticaria, angioedema and anaphylaxis) and type IV hypersensitivity reactions (e.g., inflammatory reactions in the site of injection, morbilliform and erythema multiforme-like rashes) are the most commonly observed; pityriasis rosea-like reactions, herpes zoster reactivations and functional angiopathies (e.g., chilblain-like lesions and erythromelalgia) have been also observed.^2^ Contrariwise, there are very limited data regarding autoimmune skin diseases following SARS-CoV-2 vaccines^2^ and there are only few reports of generalized morphea induced by COVID-19 mRNA vaccine.^4^

A 61-year-old Caucasian woman presented to our department with a 3-month history of ten, symmetrical, whitish, xerotic and sclerotic plaques, with a diameter ranging between 5 cm and 12 cm localized in the abdominal area, lower limbs, back and upper limbs (Figs. 1A-B and 2A). A dermoscopy of one lesion showed prominent whitish fibrotic beams (Fig. 2A). Her familiar and personal history was negative for autoimmune and chronic inﬂammatory skin disorders. She did never suffer from the Raynaud phenomenon, and she did not present sclerodactyly, with no facial involvement and no nail folds involvement. The patient referred that the first lesions appeared in the abdominal area 15 days after the first dose of Comirnaty-Pfizer® SARS-CoV-2 vaccine and lesions grew in number and size 15 days after the second dose of the vaccine. The cutaneous lesions first arose as erythematous and itchy plaques, becoming subsequently whitish and sclerotic. A cutaneous biopsy of one lesion on the abdomen was performed. The histology showed thickening and hyalinization of connective tissue of the deep dermis and subcutaneous fat, with atrophy of adnexal structures, increased fibroblasts, and dense collagens through the deep dermis (Fig. 2B). Laboratory investigations showed the presence of antinuclear antibodies (1:160 in homogeneous pattern), presence of antibodies anti-receptor-binding domain SARS-CoV-2 at 113 U/mL (normal values: <80 U/mL), while antibodies to extractable nuclear antigens were negative. According to the clinical and histological correlation, a final diagnosis of Generalized Morphea (GM) was performed and a therapy with clobetasol propionate 0.05% cream was started in association with a systemic treatment consisting in methotrexate 7.5 mg per week, with a single dose of 5 mg of folic acid once per week.

**Figure 1:**
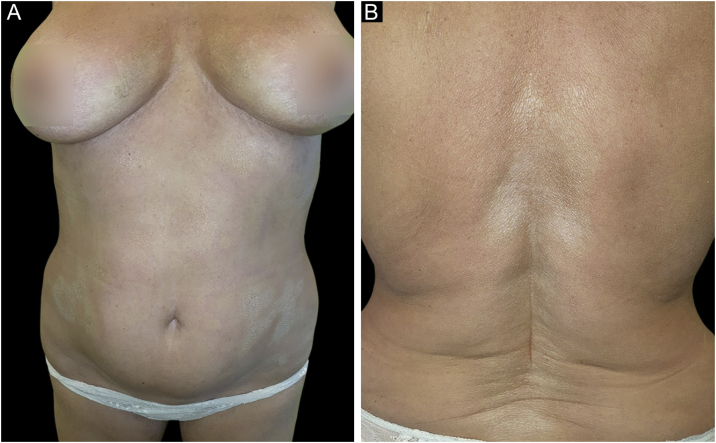
(A) Symmetrical, whitish, ivory, xerotic and sclerotic plaques, with a diameter ≥3 cm. (B) Whitish and sclerotic skin plaque with a “cigarette-paper” appearance on the patient's back.

**Figure 2:**
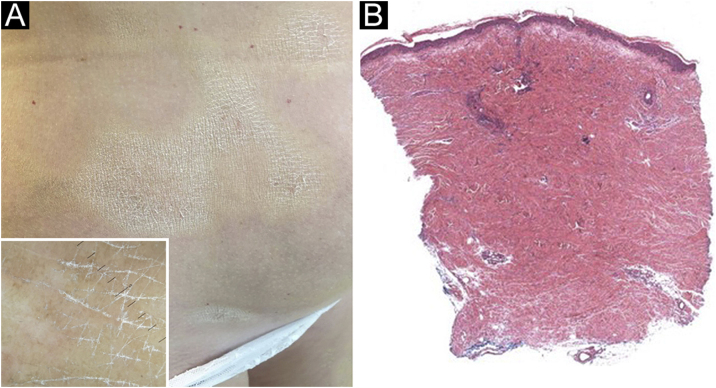
(A) Whitish and sclerotic plaques localized in abdomen and groin. Insert left: dermoscopy showed prominent whitish fibrotic beams and loss of hair in the involved area. (B) Histology showed a thickening and hyalinization of connective tissue of deep dermis and subcutaneous fat, with atrophy of adnexal structures, increased fibroblasts and dense collagens through the deep dermis (Hematoxylin & eosin, 50×).

GM is identified by more than four plaques, at least 3 cm, that involves two or more anatomical regions and differs from scleroderma by the absence of Raynaud phenomenon, sclerodactyly, facial involvement, nail fold involvement, and specific autoantibodies.^3^ In this case, the onset of GM may be justified by the fact that vaccines can occasionally cause a new onset of ﬂare of autoimmune-mediated diseases. Indeed, the spike protein of the SARS-CoV-2 vaccine shares genetic similarities with human proteins, being an important factor that can trigger autoimmune diseases after vaccination due to molecular mimicry and generation of autoreactive lymphocytes.^2^ At the same time, although the onset of cutaneous diseases after COVID-19 vaccines can highlight a causality association, the very large number of cases receiving SARS-CoV-2 vaccines during the last months may induce biases,^2^ associating with vaccines also cutaneous manifestations that could have occurred independently. In conclusion, this is one of the few cases of GM arising after the SARS-CoV-2 vaccine and further cases are needed to better investigate a possible association of GM with this vaccination. Reporting potential side effects of COVID-19 vaccines is important for daily clinical practice; at the same time, to date, COVID-19 vaccines maintain a high safety profile, and accordingly, the population should not be discouraged from vaccinating.

## Financial support

None declared.

## Authors’ contributions

Giovanni Paolino: Study concept and design; intellectual participation in the propaedeutic and/or therapeutic conduct of the studied cases; writing of the manuscript or critical review of important intellectual content; final approval of the final version of the manuscript.

Matteo Ricardo Di Nicola: Writing of the manuscript or critical review of important intellectual content; final approval of the final version of the manuscript.

Nathalie Rizzo: Study concept and design; intellectual participation in the propaedeutic and/or therapeutic conduct of the studied cases; final approval of the final version of the manuscript.

Santo Raffaele Mercuri: Study concept and design; intellectual participation in the propaedeutic and/or therapeutic conduct of the studied cases; final approval of the final version of the manuscript.

Nathalie Rizzo and Santo Raffaele Mercuri share the co-last authorship.

## Conflicts of interest

None declared.
